# Preconception diet in adolescence and its association with hypertensive disorders of pregnancy and preterm birth. Results from The HUNT study

**DOI:** 10.1017/S0007114524000746

**Published:** 2024-04-18

**Authors:** Andrew Keith Wills, Elisabet Rudjord Hillesund, Wendy Van Lippevelde, Mary Barker, Frøydis Nordgård Vik, Nina Cecilie Øverby

**Affiliations:** 1 Centre for Lifecourse Nutrition, Department of Nutrition and Public Health, University of Agder, Postboks 422, 4604 Kristiansand, Norway; 2 Unit Consumer Behaviour; Department of Marketing, Innovation and Organisation; Faculty of Economics and Business Administration; Ghent University; Tweekerkenstraat 2, 9000 Ghent, Belgium; 3 School of Health Sciences, Faculty of Environmental and Life Sciences and MRC Lifecourse Epidemiology Centre, Faculty of Medicine, University of Southampton, UK

**Keywords:** Preconception diet, pregnancy complications, preterm birth, The Hunt study

## Abstract

Our aim was to estimate associations of adolescent dietary patterns and meal habits with hypertensive disorders of pregnancy (HDP) and preterm birth. We used data from a prospective cohort study (Norwegian Young-HUNT1) where dietary information was collected during adolescence and pregnancy outcomes were obtained through record linkage to the Norwegian national birth registry. The outcomes were HDP, hypertension, preeclampsia/eclampsia, and preterm birth in the first pregnancy and in any pregnancy. Diet was self-reported from validated questionnaires and exposures were dietary indexes (healthy; unhealthy; fruit and vegetable; fibre index) and meal habits. Recruitment took place in schools. Eligible participants were females aged 13-19 years at the time of dietary assessment with a subsequent singleton pregnancy (n=3622). Women who reported a higher fibre intake in adolescence had a lower risk of pre-eclampsia in the first pregnancy (RR: 0.84; 95% CI: 0.7-1.0) although this was weaker in sensitivity analyses. Regular meal habits in mid-adolescence (aged 13-15y), particularly breakfast and lunch, were weakly associated with a lower risk of hypertension in pregnancy. Our results are the first to indicate an association between aspects of diet and dietary behavior in mid-adolescence and subsequent HDPs. More evidence is needed from larger studies to replicate the results and from alternative study-designs to disentangle causality.

## Abbreviations

HDP: Hypertensive disorders of pregnancy
GH: Gestational hypertension
DASH: Dietary Approaches to Stop Hypertension
HUNT: Trøndelag Health Study
HBSC: Health Behaviour of School-aged Children study
MBRN: Norwegian Medical Birth registry
WHO: World Health Organisation
BMI: Body mass index

## Introduction

Maternal or fetal complications occur in approximately 10-15% of pregnancies, yielding higher morbidity and mortality for mother and child^(1)^. Among these are preterm delivery and hypertensive disorders of pregnancy (HDP) such as gestational hypertension (GH), pre-eclampsia and eclampsia. Globally, the incidence of HDP has increased from 16.3 million in 1990 to 18.1 million in 2019 ^(2)^, while approximately 15 million newborns are delivered prematurely each year ^(3)^.

Part of this burden could be prevented through a healthier diet ^(4)^. For example, a recent meta-analysis of maternal diet during pregnancy and pre-eclampsia, showed that greater adherence to a healthier diet, as reported in observational studies, was associated with a 21% lower odds of preeclampsia ^(5)^. Higher adherence to a healthier diet during pregnancy has also been associated with lower risk of GH ^(6)^, and preterm delivery ^(7, 8)^. One study showed a relationship between salt intake and HDP, but not adherence to the healthier DASH diet (Dietary Approaches to Stop Hypertension) ^(9)^. With the exception of preterm birth, randomised controlled trials targeting dietary behavior during pregnancy have generally been unsuccessful in decreasing the risk of pregnancy complications ^(10–15)^. The discord between observational and experimental studies may in part reflect the presence of other more sensitive periods before conception when diet has a stronger effect. Stephenson and colleagues (2019) ^(16)^ proposed three preconception phases: (i) the biological perspective – the days to weeks before the embryo development (i.e., the periconceptual phase); (ii) the individual perspective – a conscious intention to conceive, typically weeks to months before the pregnancy occurs; and (iii) the public health perspective – longer periods of months or years with the possibility to address preconception risk factors such as diet. There is some support that diet during these windows may be important for HDP and preterm outcomes. For example, two studies that ascertained pre-pregnancy diet retrospectively before week 20, showed a lower risk of preterm birth among mothers reporting healthier pre-pregnancy diets ^(17, 18)^. We found only two cohort studies that assessed diet prospectively. The Australian Longitudinal Study on Women’s health reported a reduced risk of HDP and preterm birth in those with a more Mediterranean style and vegetable dietary pattern ^(19, 20)^, while the Nurses’ Health Study reported a lower risk of preeclampsia with higher adherence to dietary recommendations ^(21)^ and higher risk of HDP with higher pre-pregnancy intake of trans fatty acids ^(22)^. In both studies, diet was assessed at age 25 or older and up to 9 years before pregnancy.

Adolescence may be a sensitive period for diet and maternal health given the timing of female reproductive development and the increased demands for energy and nutrients to account for rapid physical growth and physiological, psychosocial, and cognitive development ^(23)^. It also coincides with more autonomy regarding lifestyle ^(24)^ which may track into adulthood with indirect effects on maternal health. However, to date no studies have explored the link between preconception diet measured in adolescence and pregnancy outcomes ^(16)^ and few cohorts have prospective dietary data collected in adolescence that can be linked to registry data containing information on pregnancy and birth outcomes. To address this, we use data from the Norwegian Young-HUNT1 Study to assess whether dietary patterns and meal eating habits during adolescence are associated with HDP and preterm birth.

## Methods

### Population and design

The Young-HUNT1 Study is the adolescent part of the Trøndelag Health Study (HUNT), a large population-based health study in the county of Nord-Trøndelag, Norway ^(25)^. Nord-Trøndelag is now part of Trøndelag county and is broadly representative of Norway with respect to economy, industry, income, age distribution, morbidity and mortality ^(25, 26)^. We used data from the Young-HUNT1 survey that took place in 1995-1997. This includes dietary and anthropometric assessment during adolescence ^(25)^ and a record linkage follow-up that captures most pregnancies of the original cohort. The study adheres to the Helsinki Declaration and was approved by the Norwegian Data Inspectorate, the Regional and National Committees for Medical and Health Research Ethics in Norway and the Norwegian Directorate of Health. Additional consent for this specific analysis was provided by the Central Regional Committee for Medical and Health Research Ethics in Norway (Reference: 2017/1220/REK midt).

Young-HUNT1 participants were recruited via schools. Principals of all the 66 schools in the county gave written consent for their school’s participation. The participation rate was 88% (n=8,980/10,202). For our investigation, eligible participants were girls and young women aged 13-19 years at the time of dietary assessment (i.e., to capture adolescent diet) with a subsequent singleton pregnancy (n=3622). Women with chronic hypertension prior to pregnancy were excluded since our interest was in incident cases (n=17).

### Dietary exposures

#### Data Collection

The dietary questionnaire was completed by pupils during school hours under assessment conditions. Specially trained nurses visited the schools to perform the anthropometric assessments using standardized protocols and equipment. Pupils absent on the day of the questionnaire were asked to complete it during the nurse visit day. Adolescents identified by the county records as out of school were invited to participate in the study by post. Here, the questionnaire was included with an invite to attend the clinical part of the study at one of the study sites for the adult part of the HUNT1 study ^(25)^.

The dietary and meal variables in the Young-HUNT1 studies were based on those assessed in the Health Behaviour of School-aged Children (HBSC) study where they were found to be reliable and valid ^(27, 28)^. Zero imputation (i.e., assumption of no consumption) was used for food and meal items that were left blank in participants who filled in more than half of those question items (1.9%).

#### Derivation of exposures

Dietary patterns were assessed using the self-reported question items: ‘How often do you drink or eat the things listed below?’ (never, < weekly, every week but not every day, once a day, and more than once a day). This was converted to number of servings per week (0=never, seldom=0.5, every week but not every day=3.5, once a day=7, 14=more than once a day). From this we computed four indexes as dietary exposures in this study. These were i) a fruit and vegetable index (calculated as the sum of fruit and vegetable intake; range: 0 to 28), ii) a fiber index (the sum of fruit, vegetable, and whole grain bread intake; range: 0 to 42), iii) a healthy diet index (the sum of fruit, vegetable, whole grain bread, whole milk, and low fat milk intake; range: 0 to 70), and iv) an unhealthy diet index (i.e., sum of soft drinks, crisps, sweets, fast food; range: 0 to 56). The use of consumption frequencies of fruit, vegetables, whole grain bread, whole milk, low fat milk, sugar sweetened soft drinks, potato chips (crisps), candy, chocolate and other sweets, and fast food as indicators of either healthy or unhealthy dietary patterns is well established ^(29, 30)^. Meal patterns were assessed using questions that asked how often the participant usually ate breakfast, lunch, and dinner (every day, 4-6 times/week, 1-3 times/week, seldom, never). These were dichotomized into daily consumption (every day) versus less than daily consumption.

### Pregnancy outcomes

Information on pregnancy outcomes was extracted through record linkage with the Norwegian Medical Birth registry (MBRN) and includes all births up to and including 2017. All live births and stillbirths in Norway from the 16th week of gestation (12th week since 2002) are notified to the MBRN on a standardised form completed by the attending midwife or obstetrician. Information on the mother’s health before and during pregnancy, including chronic diseases and complications during pregnancy and delivery are also collected. Antenatal data are brought to the birth clinic on a standardised form by the woman at the time of delivery (includes records of blood pressure, and urinary tests) and transferred to the birth notification form. Missing information on this antenatal form is collected by interview. MBRN variables have been validated against patient records and found to be satisfactory ^(31)^.

In the birth notification form from 1967 to 1998 pregnancy complications were reported in free text. Since 1999, pre-eclampsia has been notified to MBRN by marking one or more of the following tick boxes on the MBRN notification form: “pre-eclampsia, mild”, “pre-eclampsia, severe”, and “pre-eclampsia, before 34 weeks”. In addition, the form includes tick boxes for “eclampsia”, as well as for “gestational hypertension (without proteinuria)” and “pre-existing hypertension”. Free text information is also received which is coded at the MBRN using the International Classification of Diseases, 10th Revision (from 1999) ^(7, 31, 32)^. This information was used to classify each pregnancy as: normotensive, gestational hypertension or preeclampsia/eclampsia. Women with either gestational hypertension or preeclampsia/eclampsia were also classified as having hypertensive disorders of pregnancy, abbreviated HDP hereon in ^(33)^.

To estimate gestational length, the MBRN used the mother’s self-reported first day of last menstrual period until 1997, then ultrasound-based dates thereon in if available, and the first day of the last menstrual period if not. Preterm birth was defined as delivery <37 weeks of gestation ^(31)^.

### Potential confounders

Dietary patterns and habits are associated with a range of other physiological, metabolic and socio-cultural factors that may be directly and indirectly linked to pregnancy outcomes. We thus extracted information on a set of potential confounders to allow us to assess the extent of confounding and whether any observed association with the preconception dietary exposures may reflect a non-causal association through other causes. The following information was collected at the time of diet assessment using self-reported questionnaires (and coded for analysis)- education plans (higher education such as university/college; no higher education), chewing tobacco or snus (ever; never), smoking (ever; never), and alcohol use (ever; never). WHO BMI for age z-scores (de Onis et al. 2007) were derived using the weight and height measurements collected by public health nurses at schools, and measured using nationally standardised protocols ^(25)^. Maternal age at birth (years) and smoking at the start of the pregnancy (yes; no) ^(16)^ were obtained from the MBRN and were used as additional markers of potential socio-demographic confounding. HDP is associated with other pregnancy complications such as gestational diabetes, this information was extracted from the MBRN record of the mother.

### Main analyses

All variables for the eligible and included mothers were described alongside those eligible but excluded due to missing data on at least one variable. Potential confounders were summarised according to exposure groups defined by tertiles of the healthy eating index and typical meal patterns.

The outcomes were HDP, hypertension, preeclampsia/ eclampsia, and preterm birth. HDP and preterm birth were modelled using logistic regression, hypertension and preeclampsia were modelled as separate categories using multinomial logistic regression. The exposures were the adolescent diet indexes (healthy; unhealthy; fruit and vegetable; fibre) and meal habits (daily breakfast; daily lunch; daily dinner). Two risk windows were defined, (a) the risk in the first pregnancy; and (b) the risk in any pregnancy. The latter is an estimate of the cumulative reproductive risk since we have information on all pregnancies up to ages 35 to 41 years (depending on the age of the mother when the birth records were linked in 2017), which for most women would capture their entire reproductive history.

Crude and adjusted associations were estimated and compared to assess potential confounding. Since educational plans, experience of smoking, snus or alcohol is age-dependent, interactions with age at assessment were included. To improve causal inference, an additional set of models were estimated that included an interaction with age at diet assessment (split by tertiles). Since the age at diet assessment was pseudo-random and uniform from 13-19y due to the study design, confounding of the diet-outcome relationship is likely to be similar across ages. Thus, any qualitatively different pattern in the strength of the diet-outcome association across age might reflect a pathway that is not explained by residual confounding. For example, a stronger association in the earlier period of adolescence examined here might indicate a sensitive period for dietary exposure, whereas a stronger association in the later periods might reflect an association due to tracking of diet.

### Sensitivity analyses

To assess the extent and robustness of our main findings to bias we conducted sensitivity analyses. First, we excluded women who had gestational diabetes since this is also associated with HDP, results (available on request) were similar so we discuss this no further. Second, to assess potential bias caused by missing data, we compared the unadjusted estimates in the complete cases with those using all available data.

Since information on smoking status at the start of pregnancy was missing for many cases (n=435, 14.3%) we omitted this variable from the adjusted models, however, we justified this by comparing estimates with and without adjustment for smoking at the start of pregnancy in the same sample as a check for residual confounding and findings were unaltered (results available on request). STATA 17.0 was used for all analyses.

## Results

### Sample description

**Figure 1:**
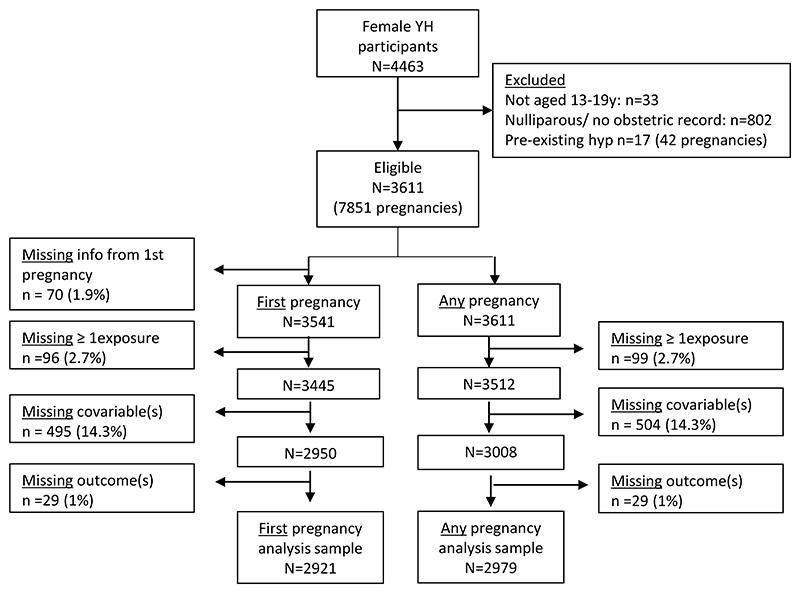
Flow chart to illustrate the sample selection and reasons for exclusions and missing data.

Figure 1 shows how the analysis sample was selected. From 4463 girls and young women recruited into Young-HUNT1, 802 (18%) were nulliparous or had no obstetric record at the time of record linkage (aged 35-41 years). More than 80% of those eligible were included in the analysis. The main reason for exclusion was missing covariable information.

Table 1 shows the characteristics of those eligible and included in the analysis. At diet assessment the average age was 16 years, BMI was slightly higher than the WHO growth reference, the majority had tried alcohol and smoking and a small proportion reported use of snus. Those excluded due to missing data were on average slightly older at point of dietary assessment (+0.5y). Since diet, education plans and experience of smoking and alcohol are age-dependent (see supplementary information Table S1), these characteristics were also different among those with missing data.

### Associations between adolescent exposures and potential confounders:

Table 2 shows that those with healthier diets during adolescence were on average more likely to have plans for higher education and less likely to have ever smoked or used alcohol or chewed tobacco. Their first pregnancies also occurred on average at a slightly older age and they were less likely to be smoking at conception. Similar patterns were seen among those who reported eating lunch daily during adolescence (see supplementary information Table S2)

### Associations among dietary and meal exposures

Supplementary information Tables S3 & S4 show the associations between the dietary and meal habit exposure variables. The healthy diet, fruit and vegetable, and fibre indexes shared strong correlations (range of r: 0.74 - 0.89), while the unhealthy index shared little correlation (range of r: -0.11 to -0.05). The daily meal habit exposures were weakly concordant with each other (range of kappa: 0.21 to 0.37). On average, adolescents who reported regular meal habits also had higher scores on the healthy diet, fruit and vegetable, and fibre indexes.

### Associations between adolescent diet and pregnancy outcomes

**Figure 2:**
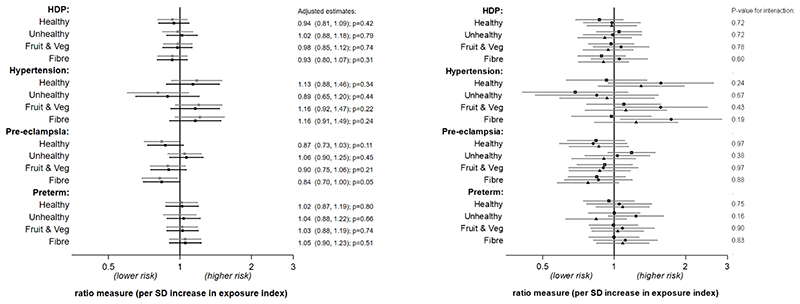
Association between diet indexes and hypertensive disorders and preterm birth in the first pregnancy (n=2921). Estimates are odds ratios for HDP and preterm birth outcome (logit models) and relative risk ratios for hypertension and pre-eclampsia (multinomial logit models). Left plot: no age interaction (grey: crude; black: adjusted*). Right plot: unadjusted associations stratified by age of diet assessment (spit by tertiles: squares 13-15.1y; circles 15.2-16.9y; triangles 17-19y). *adjusted for age, WHO BMI z-score, alcohol (ever), smoking (ever), snus use (ever) and education plans at diet assessment, and maternal age at birth.

Figure 2 shows the associations between the adolescent dietary pattern indexes and outcomes in the first pregnancy while figure S1 (see supplementary information) shows the same results but for risk in any pregnancy. There was a weak suggestion of an association between adolescent fibre intake and pre-eclampsia - an SD higher fibre index score was associated with a 16% reduction in risk of pre-eclampsia in the first pregnancy. This association was weaker when examining risk in any pregnancy (-12%, p=0.10) and was attenuated in the sensitivity analyses that included all available data in the unadjusted model (risk ratio in complete cases: 0.82, p=0.02; risk ratio using all available data: 0.87, p=0.08 – see supplementary information figure S2). There was little evidence of any other association between the diet indexes and outcomes. Likewise, there was little suggestion of any difference or any consistent qualitative pattern in the associations stratified by the age at which diet was assessed. Adjusting for potential confounders generally attenuated estimates slightly towards the null.

**Figure 3:**
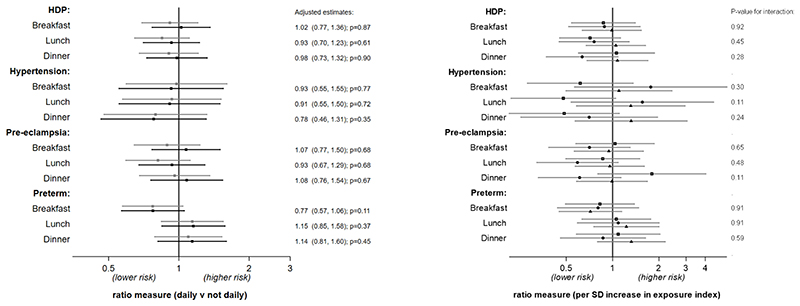
Association between meal patterns and hypertensive disorders and preterm birth in the first pregnancy (n=2921). Estimates are odds ratios for HDP and preterm birth outcome (logit models) and relative risk ratios for hypertension and pre-eclampsia (multinomial logit models). Left plot: no age interaction (grey: crude; black: adjusted*). Right plot: unadjusted associations stratified by age of diet assessment (spit by tertiles: squares 13-15.1y; circles 15.2-16.9y; triangles 17-19y). *adjusted for age, WHO BMI z-score, alcohol (ever), smoking (ever), snus use (ever) and education plans at diet assessment, and maternal age at birth.

The results for adolescent meal patterns are presented in the same format for the outcomes in the first pregnancy (figure 3) and for the outcomes in any pregnancy (see supplementary information figure S3). There was little evidence to support an effect of meal patterns on HDP or preterm birth outcomes in the unstratified analyses. However, in the age-stratified analysis, there was a weak suggestion that the earlier period of adolescence examined (13-15y) might be a sensitive period with regular lunch associated with a lower risk of later hypertension. In the sensitivity analysis using all available data, adolescents aged 13-15y that reported consuming regular breakfast also had a lower risk of hypertension in pregnancy (see supplementary information figure S4).

## Discussion

### Summary of findings

This is the first study to estimate the association between adolescent diet and meal patterns and risk of hypertensive disorders in pregnancy and preterm birth. We found a slightly lower risk of pre-eclampsia in the first pregnancy among those women who had reported a higher fibre intake during adolescence, although this result was somewhat dependent on the reproductive window examined and was not robust in sensitivity analyses to explore bias by missing data. Meal habits showed an age-dependent association with hypertension − those reporting eating regular meals from 13-15y, particularly breakfast and lunch, had a lower risk of hypertension.

### Comparison with other studies and mechanisms related to fibre and preeclampsia

The associations between fibre intake and preeclampsia are in line with Shoenaker et al (2015) who reported inverse associations between Mediterranean-style dietary pattern (characterized by vegetables, legumes, nuts, tofu, rice, pasta, rye bread, red wine, and fish) and risk of developing HDPs confined to GH (quartile 4 compared with quartile 1: RR, 0.58; 95% CI, 0.42, 0.81) ^(19)^. It also concords with Hillesund et al. 2014 who reported an association between a healthy pre-pregnancy diet score and preeclampsia, the score reflecting fibre intake especially from fruit and vegetables ^(8)^.

Fibre index in the present study was derived from frequency of fruit, vegetable and whole grain bread intake. So what does the fibre index represent in terms of nutritional value of relevance to preeclampsia? A higher intake of dietary fiber aids in weight maintenance. Fibre also lowers serum triglycerides and low densitiy lipoprotein-cholesterol, known to increase the risk of hypertension and cardiovascular disease. A narrative review by Perry et al. (2022) highlighted that consuming a high-fibre diet (25-30 g/day) may reduce the risk of preeclampsia through beneficial effects on inflammation, blood lipids and blood pressure ^(34)^. Dietary fibre intake is also a marker of foods with high nutritional value, with wholegrains containing magnesium, selenium, folate and other B-vitamins. The other food group rich in fibre is fruits and vegetables with ample amounts of antioxidants and antiinflammatory potential. Dietary fibre also acts as a prebiotic through promoting the growth and abundance of beneficial large intestine bacteria ^(35)^. Intake of probiotics during pregnancy has been shown to reduce the risk of preeclampsia ^(36)^.

### Comparison with other studies and mechanisms related to meal habits and gestational hypertension

Those reporting eating regular meals in mid-adolescence (13-15y), particularly breakfast and lunch, had a lower risk of hypertension compared to those with a similar exposure in later periods of adolescence. No other studies have investigated this association. What does eating breakfast and lunch daily versus less often imply? In Norway, breakfast and lunch typically include wholegrain bread or cereal which are main sources of dietary fibre in the diet. In addition, regularly eating breakfast and lunch provides metabolic fuel and critical nutrients for growth and accrual of body mass in this life stage. Regular meals may also prevent overeating and obesity although the evidence on causality of this association is inconsistent ^(37)^.

The observed association between dietary behaviour (fibre intake, and daily breakfast and lunch) among 13-15 year olds and reduced odds of preeclampsia and GH, respectively, many years later, could be explained by habits in early adolescence tracking into adulthood. Another explanation of the finding confined to those who reported diet at 13-15 years could be that mid-adolescence is a biologically sensitive phase in girls regarding meal habits and related diet quality and later reproductive health.

### Strengths and limitations

Strengths of our study include the unique sample containing over 7000 births with prospectively measured diet and a long follow up, enabling assessment of diet in adolescence in relation to not only first pregnancy but also the entire reproductive window for most mothers. Further, the diagnosis of preeclampsia and GH was based on hospital medical records from the Medical Birth Registry of Norway and is objectively verified. Disentangling cause from association is notoriously troublesome in observational nutrition research, in this respect, our analysis stratified by age of exposure offered an additional test to help triangulate our findings. Nonetheless, there are still assumptions such as measurement error to be similar across ages that would affect the validity of these comparisons.

Among limitations are the quality of the dietary data, which lacks resolution due to the small number of food items included in the questionnaire. Information on fish, meat, fats, and more information on fruits, berries and vegetables variety are also important aspects to consider. The highest obtainable frequency of intake was twice or more per day. In addition to possible dilution of associations by random error, there is also likely to be differential measurement error caused by under-reporting of unhealthy dietary components by more at risk groups such as those with high BMI ^(38).^

## Conclusions

Our results are the first to indicate an association between aspects of diet and dietary behavior in mid adolescence and subsequent HDPs. While a healthy diet throughout life is well advocated, a better understanding of sensitive windows for dietary intervention in relation to aspects of maternal health is still needed. With regards to our findings, more evidence is needed to replicate the results and to disentangle causality. Such studies will need larger samples with more detailed measures of diet and confounders, and will exploit alternative designs with different sources of bias in order to triangulate findings.

## Supplementary Material

Supplementary Information

## Figures and Tables

**Table 1:** Participant characteristics in those eligible and included in the analyses of first pregnancies (n=2921*) and those eligible but excluded from analyses due to missing data.

	Eligible & included		Eligible but missing data		p-value†
	N	mean (SD) or %	n	Mean (SD) or %	
At diet assessment (adolescence)					
Age (years)	2921	16.0 (1.7)	690	16.5 (2.2)	<0.001
WHO BMI (z-score)	2921	0.17 (0.89)	377	0.13 (0.93)	0.37
Education plans (Higher education)	2921	34%	567	34%	0.9
Smoking (ever)	2921	59%	623	61%	0.29
Alcohol (ever)	2921	84%	570	88%	0.021
Snus use (ever)	2921	4.8%	639	6.0%	0.23
At first pregnancy					
Maternal age at birth (years)	2921	25.9 (4.4)	690	25.4 (4.8)	0.005
Smoking at beginning of pregnancy (yes)	2471	16.6%	526	18.4%	0.32
Dietary exposures (adolescence) ‡					
Healthy foods index [0-70]	2921	31 (12)	690	27 (15)	<0.001
Fiber index [0-42]	2921	21 (10)	690	18 (11)	<0.001
Fruit & Vegetable Index [0-28]	2921	12 (7)	690	10 (8)	<0.001
Unhealthy foods Index [0-56]	2921	11 (6)	690	10 (7)	0.11
Daily breakfast (yes)	2921	64%	629	63%	0.6
Daily lunch (yes)	2921	61%	620	59%	0.31
Daily dinner (yes)	2921	71%	629	70%	0.5
Outcomes (first pregnancy)					
Hypertensive Disorder of pregnancy:					
Normotensive	2921	92%		94.5%	
Hypertensive		2.36%	620	1.61%	0.10
Pre-eclampsia/ Eclampsia		5.61%		3.87%	
Preterm delivery	2921	6.50%	579	8.46%	0.09

* Based on analysis of first pregnancies

† p-values are from a t-test for continuous variables and a chi-squared test for categorical variables

‡ the values for the dietary exposures are obtained by summing the frequency of intake of components that comprise each index

**Table 2:** Participant characteristics according to percentile-based categories of the Healthy food index (dietary exposure)

	Healthy diet index (adolescence)			
	Lowest third (<24) n=	Middle third (24 to 35) n=	Highest third (>35) n=	p-value
At diet assessment:				
Age (years)	16.1	16.1	15.9	0.01
WHO BMI (z-score)	0.20	0.18	0.15	0.46
Education plans (Higher education)	29.3%	34.7%	37.8%	0.001
Smoking (ever)	67.1%	57.7%	51.8%	<0.001
Alcohol (ever)	87.2%	85.7%	79.9%	<0.001
Snus use (ever)	6.0%	3.6%	5.2%	0.035
At birth*:				
Maternal age (years)	25.4	25.9	26.4	<0.001
Smoking at beginning of pregnancy (yes)	20.0%	17.1%	13.0%	0.001

* Characteristics at first included pregnancy

**Table 1:** STROBE-nut: An extension of the STROBE statement for nutritional epidemiology Lachat C et al. (2016) STrengthening the Reporting of OBservational studies in Epidemiology – Nutritional Epidemiology (STROBE-nut): an extension of the STROBE statement. Plos Medicine 13(6) http://dx.doi.org/10.1371/journal.pmed.1002036_pdf or online version.

Item	Item nr	STROBE recommendations	Extension for Nutritional Epidemiology studies (STROBE-nut)	Reported on page #
**Title and abstract**	1	(a) Indicate the study’s design with a commonly used term in the title or the abstract. (b) Provide in the abstract an informative and balanced summary of what was done and what was found.	**nut-1** State the dietary/nutritional assessment method(s) used in the title, abstract, or keywords.	**2**
**Introduction**				
Background rationale	2	Explain the scientific background and rationale for the investigation being reported.		**3**
Objectives	3	State specific objectives, including any prespecified hypotheses.		**4**
**Methods**				
Study design	4	Present key elements of study design early in the paper.		**4**
Settings	5	Describe the setting, locations, and relevant dates, including periods of recruitment, exposure, follow-up, and data collection.	**nut-5** Describe any characteristics of the study settings that might affect the dietary intake or nutritional status of the participants, if applicable.	**4**
Participants	6	(a) Cohort study—Give the eligibility criteria, and the sources and methods of selection of participants. Describe methods of follow-up. Case-control study—Give the eligibility criteria, and the sources and methods of case ascertainment and control selection. Give the rationale for the choice of cases and controls. Cross-sectional study—Give the eligibility criteria, and the sources and methods of selection of participants. (b) Cohort study—For matched studies, give matching criteria and number of exposed and unexposed. Case-control study—For matched studies, give matching criteria and the number of controls per case.	**nut-6** Report particular dietary, physiological or nutritional characteristics that were considered when selecting the target population.	**4**
Variables	7	Clearly define all outcomes, exposures, predictors, potential confounders, and effect modifiers. Give diagnostic criteria, if applicable.	**nut-7.1** Clearly define foods, food groups, nutrients, or other food components. **nut-7.2** When using dietary patterns or indices, describe the methods to obtain them and their nutritional properties.	**5; 5**
Data sources - measurements	8	For each variable of interest, give sources of data and details of methods of assessment (measurement).Describe comparability of assessment methods if there is more than one group.	**nut-8.1** Describe the dietary assessment method(s), e.g., portion size estimation, number of days and items recorded, how it was developed and administered, and how quality was assured. Report if and how supplement intake was assessed. **nut-8.2** Describe and justify food composition data used. Explain the procedure to match food composition with consumption data. Describe the use of conversion factors, if applicable. **nut-8.3** Describe the nutrient requirements, recommendations, or dietary guidelines and the evaluation approach used to compare intake with the dietary reference values, if applicable. **nut-8.4** When using nutritional biomarkers, additionally use the STROBE Extension for Molecular Epidemiology (STROBE-ME). Report the type of biomarkers used and their usefulness as dietary exposure markers. **nut-8.5** Describe the assessment of nondietary data (e.g., nutritional status and influencing factors) and timing of the assessment of these variables in relation to dietary assessment. **nut-8.6** Report on the validity of the dietary or nutritional assessment methods and any internal or external validation used in the study, if applicable.	**5**
Bias	9	Describe any efforts to address potential sources of bias.	**nut-9** Report how bias in dietary or nutritional assessment was addressed, e.g., misreporting, changes in habits as a result of being measured, or data imputation from other sources	**5; 7; 8**
Study Size	10	Explain how the study size was arrived at.		
Quantitative variables	11	Explain how quantitative variables were handled in the analyses. If applicable, describe which groupings were chosen and why.	**nut-11** Explain categorization of dietary/nutritional data (e.g., use of N-tiles and handling of nonconsumers) and the choice of reference category, if applicable.	**5**
Statistical Methods	12	(a) Describe all statistical methods, including those used to control for confounding (b) Describe any methods used to examine subgroups and interactions. (c) Explain how missing data were addressed. (d) Cohort study—If applicable, explain how loss to follow-up was addressed. Case-control study—If applicable, explain how matching of cases and controls was addressed. Cross-sectional study—If applicable, describe analytical methods taking account of sampling strategy. Describe any sensitivity analyses.	**nut-12.1** Describe any statistical method used to combine dietary or nutritional data, if applicable. **nut-12.2** Describe and justify the method for energy adjustments, intake modeling, and use of weighting factors, if applicable. **nut-12.3** Report any adjustments for measurement error, i.e,. from a validity or calibration study.	**7; 8**
**Results**				
Participants	13	(a) Report the numbers of individuals at each stage of the study—e.g., numbers potentially eligible, examined for eligibility, confirmed eligible, included in the study, completing follow-up, and analyzed. (b) Give reasons for non-participation at each stage. (c) Consider use of a flow diagram.	**nut-13** Report the number of individuals excluded based on missing, incomplete or implausible dietary/ nutritional data.	Figure 1; **p9**
Descriptive data	14	(a) Give characteristics of study participants (e.g., demographic, clinical, social) and information on exposures and potential confounders (b) Indicate the number of participants with missing data for each variable of interest (c) Cohort study—Summarize follow-up time (e.g., average and total amount)	**nut-14** Give the distribution of participant characteristics across the exposure variables if applicable. Specify if food consumption of total population or consumers only were used to obtain results.	**P9**; suppl tables 2,3,4
Outcome data	15	Cohort study—Report numbers of outcome events or summary measures over time. Case-control study—Report numbers in each exposure category, or summary measures of exposure. Cross-sectional study—Report numbers of outcome events or summary measures.		**9**, Table 1
Main results	16	(a) Give unadjusted estimates and, if applicable, confounder-adjusted estimates and their precision (e.g., 95% confidence interval). Make clear which confounders were adjusted for and why they were included. (b) Report category boundaries when continuous variables were categorized. (c) If relevant, consider translating estimates of relative risk into absolute risk for a meaningful time period.	**nut-16** Specify if nutrient intakes are reported with or without inclusion of dietary supplement intake, if applicable.	**9, 10**
Other analyses	17	Report other analyses done—e.g., analyses of subgroups and interactions and sensitivity analyses.	**nut-17** Report any sensitivity analysis (e.g., exclusion of misreporters or outliers) and data imputation, if applicable.	**9,10**
**Discussion**				
Key results	18	Summarize key results with reference to study objectives.		11
Limitation	19	Discuss limitations of the study, taking into account sources of potential bias or imprecision. Discuss both direction and magnitude of any potential bias.	**nut-19** Describe the main limitations of the data sources and assessment methods used and implications for the interpretation of the findings.	**12**
Interpretation	20	Give a cautious overall interpretation of results considering objectives, limitations, multiplicity of analyses, results from similar studies, and other relevant evidence.	**nut-20** Report the nutritional relevance of the findings, given the complexity of diet or nutrition as an exposure.	**11-12**
Generalizability	21	Discuss the generalizability (external validity) of the study results.		**12**
**Other information**				
Funding	22	Give the source of funding and the role of the funders for the present study and, if applicable, for the original study on which the present article is based.		**13**
*Ethics*			**nut-22.1** Describe the procedure for consent and study approval from ethics committee(s).	**4**
Supplementary material			**nut-22.2** Provide data collection tools and data as online material or explain how they can be accessed.	
