# Preconception diet in adolescence and its association with hypertensive disorders of pregnancy and preterm birth. Results from the HUNT study

**DOI:** 10.1017/S0007114524000746

**Published:** 2024-07-14

**Authors:** Andrew Keith Wills, Elisabet Rudjord Hillesund, Wendy van Lippevelde, Mary Barker, Frøydis Nordgård Vik, Nina Cecilie Øverby

**Affiliations:** 1 Centre for Lifecourse Nutrition, Department of Nutrition and Public Health, University of Agder, Postboks 422, 4604 Kristiansand, Norway; 2 Unit Consumer Behaviour, Department of Marketing, Innovation and Organisation, Faculty of Economics and Business Administration, Ghent University, Tweekerkenstraat 2, 9000 Ghent, Belgium; 3 School of Health Sciences, Faculty of Environmental and Life Sciences and MRC Lifecourse Epidemiology Centre, Faculty of Medicine, University of Southampton, Southampton, UK

**Keywords:** Preconception diet, Pregnancy complications, Preterm birth, The Hunt study

## Abstract

Our aim was to estimate associations of adolescent dietary patterns and meal habits with hypertensive disorders of pregnancy (HDP) and preterm birth. We used data from a prospective cohort study (Norwegian Young-HUNT1) where dietary information was collected during adolescence and pregnancy outcomes were obtained through record linkage to the Norwegian national birth registry. The outcomes were HDP, hypertension, pre-eclampsia/eclampsia, and preterm birth in the first pregnancy and in any pregnancy. Diet was self-reported from validated questionnaires, and exposures were dietary indexes (healthy; unhealthy; fruit and vegetable; fibre index) and meal habits. Recruitment took place in schools. Eligible participants were females aged 13–19 years at the time of dietary assessment with a subsequent singleton pregnancy (*n* 3622). Women who reported a higher fibre intake in adolescence had a lower risk of pre-eclampsia in the first pregnancy (Relative Risk: 0·84; 95 % CI 0·7, 1·0), although this was weaker in sensitivity analyses. Regular meal habits in mid-adolescence (aged 13–15 years), particularly breakfast and lunch, were weakly associated with a lower risk of hypertension in pregnancy. Our results are the first to indicate an association between aspects of diet and dietary behaviour in mid-adolescence and subsequent HDP. More evidence is needed from larger studies to replicate the results and from alternative study designs to disentangle causality.

Maternal or fetal complications occur in approximately 10–15 % of pregnancies, yielding higher morbidity and mortality for mother and child^(1)^. Among these are preterm delivery and hypertensive disorders of pregnancy (HDP) such as gestational hypertension (GH), pre-eclampsia and eclampsia. Globally, the incidence of HDP has increased from 16·3 million in 1990 to 18·1 million in 2019^(2)^, while approximately 15 million newborns are delivered prematurely each year^(3)^.

Part of this burden could be prevented through a healthier diet^(4)^. For example, a recent meta-analysis of maternal diet during pregnancy and pre-eclampsia showed that greater adherence to a healthier diet, as reported in observational studies, was associated with a 21 % lower odds of pre-eclampsia^(5)^. Higher adherence to a healthier diet during pregnancy has also been associated with lower risk of GH^(6)^ and preterm delivery^(7,8)^. One study showed a relationship between salt intake and HDP, but not adherence to the healthier DASH diet (Dietary Approaches to Stop Hypertension)^(9)^. With the exception of preterm birth, randomised controlled trials targeting dietary behaviour during pregnancy have generally been unsuccessful in decreasing the risk of pregnancy complications^(10–15)^. The discord between observational and experimental studies may in part reflect the presence of other more sensitive periods before conception when diet has a stronger effect. Stephenson and colleagues (2019)^(16)^ proposed three preconception phases: (i) the biological perspective – the days to weeks before the embryo development (i.e. the periconceptual phase); (ii) the individual perspective – a conscious intention to conceive, typically weeks to months before the pregnancy occurs; and (iii) the public health perspective – longer periods of months or years with the possibility to address preconception risk factors such as diet. There is some support that diet during these windows may be important for HDP and preterm outcomes. For example, two studies that ascertained pre-pregnancy diet retrospectively before week 20 showed a lower risk of preterm birth among mothers reporting healthier pre-pregnancy diets^(17,18)^. We found only two cohort studies that assessed diet prospectively. The Australian Longitudinal Study on Women’s health reported a reduced risk of HDP and preterm birth in those with a more Mediterranean style and vegetable dietary pattern^(19,20)^, while the Nurses’ Health Study reported a lower risk of pre-eclampsia with higher adherence to dietary recommendations^(21)^ and higher risk of HDP with higher pre-pregnancy intake of *trans*-fatty acids^(22)^. In both studies, diet was assessed at age 25 years or older and up to 9 years before pregnancy.

Adolescence may be a sensitive period for diet and maternal health given the timing of female reproductive development and the increased demands for energy and nutrients to account for rapid physical growth and physiological, psychosocial, and cognitive development^(23)^. It also coincides with more autonomy regarding lifestyle^(24)^ which may track into adulthood with indirect effects on maternal health. However, to date no studies have explored the link between preconception diet measured in adolescence and pregnancy outcomes^(16)^, and few cohorts have prospective dietary data collected in adolescence that can be linked to registry data containing information on pregnancy and birth outcomes. To address this, we use data from the Norwegian Young-HUNT1 Study to assess whether dietary patterns and meal eating habits during adolescence are associated with HDP and preterm birth.

## Methods

### Population and design

The Young-HUNT1 Study is the adolescent part of the Trøndelag Health Study (HUNT), a large population-based health study in the county of Nord-Trøndelag, Norway^(25)^. Nord-Trøndelag is now part of Trøndelag county and is broadly representative of Norway with respect to economy, industry, income, age distribution, morbidity and mortality^(25,26)^. We used data from the Young-HUNT1 survey that took place in 1995–1997. This includes dietary and anthropometric assessment during adolescence^(25)^ and a record linkage follow-up that captures most pregnancies of the original cohort. The study adheres to the Helsinki Declaration and was approved by the Norwegian Data Inspectorate, the Regional and National Committees for Medical and Health Research Ethics in Norway and the Norwegian Directorate of Health. Additional consent for this specific analysis was provided by the Central Regional Committee for Medical and Health Research Ethics in Norway (reference: 2017/1220/REK midt).

Young-HUNT1 participants were recruited via schools. Principals of all the sixty-six schools in the county gave written consent for their school’s participation. The participation rate was 88 % (*n* 8980/10 202). For our investigation, eligible participants were girls and young women aged 13–19 years at the time of dietary assessment (i.e. to capture adolescent diet) with a subsequent singleton pregnancy (*n* 3622). Women with chronic hypertension prior to pregnancy were excluded since our interest was in incident cases (*n* 17).

### Dietary exposures

#### Data collection

The dietary questionnaire was completed by pupils during school hours under assessment conditions. Specially trained nurses visited the schools to perform the anthropometric assessments using standardised protocols and equipment. Pupils absent on the day of the questionnaire were asked to complete it during the nurse visit day. Adolescents identified by the county records as out of school were invited to participate in the study by post. Here, the questionnaire was included with an invite to attend the clinical part of the study at one of the study sites for the adult part of the HUNT1 study^(25)^.

The dietary and meal variables in the Young-HUNT1 studies were based on those assessed in the Health Behaviour of School-aged Children (HBSC) study where they were found to be reliable and valid^(27)^. Zero imputation (i.e. assumption of no consumption) was used for food and meal items that were left blank in participants who filled in more than half of those question items (1·9 %).

#### Derivation of exposures

Dietary patterns were assessed using the self-reported question items: ‘How often do you drink or eat the things listed below?’ (never, < weekly, every week but not every day, once a day and more than once a day). This was converted to number of servings per week (0 = never, seldom = 0·5, every week but not every day = 3·5, once a day = 7 and 14 = more than once a day). From this, we computed four indexes as dietary exposures in this study. These were (i) a fruit and vegetable index (calculated as the sum of fruit and vegetable intake; range: 0–28), (ii) a fibre index (the sum of fruit, vegetable and wholegrain bread intake; range: 0–42), (iii) a healthy diet index (the sum of fruit, vegetable, wholegrain bread, whole milk and low-fat milk intake; range: 0–70) and (iv) an unhealthy diet index (i.e. sum of soft drinks, crisps, sweets and fast food; range: 0–56). The use of consumption frequencies of fruit, vegetables, wholegrain bread, whole milk, low-fat milk, sugar-sweetened soft drinks, potato chips (crisps), candy, chocolate and other sweets, and fast food as indicators of either healthy or unhealthy dietary patterns is well established^(28,29)^. Meal patterns were assessed using questions that asked how often the participant usually ate breakfast, lunch and dinner (every day, 4–6 times/week, 1–3 times/week, seldom and never). These were dichotomised into daily consumption (every day) *v*. less than daily consumption.

### Pregnancy outcomes

Information on pregnancy outcomes was extracted through record linkage with the Norwegian Medical Birth Registry (MBRN) and includes all births up to and including 2017. All live births and stillbirths in Norway from the 16th week of gestation (12th week since 2002) are notified to the MBRN on a standardised form completed by the attending midwife or obstetrician. Information on the mother’s health before and during pregnancy, including chronic diseases and complications during pregnancy and delivery, are also collected. Antenatal data are brought to the birth clinic on a standardised form by the woman at the time of delivery (includes records of blood pressure and urinary tests) and transferred to the birth notification form. Missing information on this antenatal form is collected by interview. MBRN variables have been validated against patient records and found to be satisfactory^(31)^.

In the birth notification form from 1967 to 1998, pregnancy complications were reported in free text. Since 1999, pre-eclampsia has been notified to MBRN by marking one or more of the following tick boxes on the MBRN notification form: ‘pre-eclampsia, mild’, ‘pre-eclampsia, severe’ and ‘pre-eclampsia, before 34 weeks’. In addition, the form includes tick boxes for ‘eclampsia’, as well as for ‘gestational hypertension (without proteinuria)’ and ‘pre-existing hypertension’. Free text information is also received which is coded at the MBRN using the International Classification of Diseases, 10th Revision (from 1999)^(7,30,31)^. This information was used to classify each pregnancy as: normotensive, GH or pre-eclampsia/eclampsia. Women with either GH or pre-eclampsia/eclampsia were also classified as having HDP^(32)^.

To estimate gestational length, the MBRN used the mother’s self-reported first day of last menstrual period until 1997, then ultrasound-based dates thereon in if available, and the first day of the last menstrual period if not. Preterm birth was defined as delivery < 37 weeks of gestation^(32)^.

### Potential confounders

Dietary patterns and habits are associated with a range of other physiological, metabolic and sociocultural factors that may be directly and indirectly linked to pregnancy outcomes. We thus extracted information on a set of potential confounders to allow us to assess the extent of confounding and whether any observed association with the preconception dietary exposures may reflect a non-causal association through other causes. The following information was collected at the time of diet assessment using self-reported questionnaires (and coded for analysis): education plans (higher education such as university/college; no higher education), chewing tobacco or snus (ever; never), smoking (ever; never) and alcohol use (ever; never). WHO BMI for age z-scores (de Onis *et al.* 2007) were derived using the weight and height measurements collected by public health nurses at schools and measured using nationally standardised protocols^(25)^. Maternal age at birth (years) and smoking at the start of the pregnancy (yes; no)^(16)^ were obtained from the MBRN and were used as additional markers of potential sociodemographic confounding. HDP is associated with other pregnancy complications such as gestational diabetes, and this information was extracted from the MBRN record of the mother.

### Main analyses

All variables for the eligible and included mothers were described alongside those eligible but excluded due to missing data on at least one variable. Potential confounders were summarised according to exposure groups defined by tertiles of the healthy eating index and typical meal patterns.

The outcomes were HDP, hypertension, pre-eclampsia/eclampsia and preterm birth. HDP and preterm birth were modelled using logistic regression, and hypertension and pre-eclampsia were modelled as separate categories using multinomial logistic regression. The exposures were the adolescent diet indexes (healthy; unhealthy; fruit and vegetable; fibre) and meal habits (daily breakfast; daily lunch; daily dinner). Two risk windows were defined: (a) the risk in the first pregnancy and (b) the risk in any pregnancy. The latter is an estimate of the cumulative reproductive risk since we have information on all pregnancies up to ages 35–41 years (depending on the age of the mother when the birth records were linked in 2017), which for most women would capture their entire reproductive history.

Crude and adjusted associations were estimated and compared to assess potential confounding. Since educational plans, experience of smoking, snus or alcohol are age-dependent, interactions with age at assessment were included. To improve causal inference, an additional set of models were estimated that included an interaction with age at diet assessment (split by tertiles). Since the age at diet assessment was pseudo-random and uniform from 13 to 19 years due to the study design, confounding of the diet–outcome relationship is likely to be similar across ages. Thus, any qualitatively different pattern in the strength of the diet–outcome association across age might reflect a pathway that is not explained by residual confounding. For example, a stronger association in the earlier period of adolescence examined here might indicate a sensitive period for dietary exposure, whereas a stronger association in the later periods might reflect an association due to tracking of diet.

### Sensitivity analyses

To assess the extent and robustness of our main findings to bias, we conducted sensitivity analyses. First, we excluded women who had gestational diabetes since this is also associated with HDP, results (available on request) were similar, so we discuss this no further. Second, to assess potential bias caused by missing data, we compared the unadjusted estimates in the complete cases with those using all available data.

Since information on smoking status at the start of pregnancy was missing for many cases (*n* 435, 14·3 %), we omitted this variable from the adjusted models; however, we justified this by comparing estimates with and without adjustment for smoking at the start of pregnancy in the same sample as a check for residual confounding and findings were unaltered (results available on request). STATA 17.0 was used for all analyses.

## Results

### Sample description

**Fig. 1.**
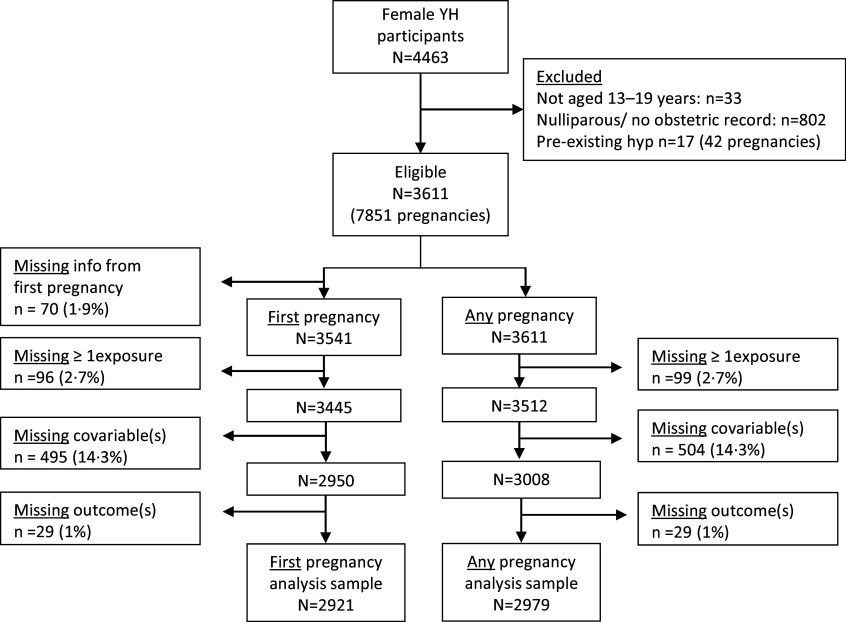
Flow chart to illustrate the sample selection and reasons for exclusions and missing data.

Figure 1 shows how the analysis sample was selected. From 4463 girls and young women recruited into Young-HUNT1, 802 (18 %) were nulliparous or had no obstetric record at the time of record linkage (aged 35–41 years). More than 80 % of those eligible were included in the analysis. The main reason for exclusion was missing covariable information.

Table 1 shows the characteristics of those eligible and included in the analysis. At diet assessment, the average age was 16 years, BMI was slightly higher than the WHO growth reference, the majority had tried alcohol and smoking, and a small proportion reported use of snus. Those excluded due to missing data were on average slightly older at point of dietary assessment (+0·5 years). Since diet, education plans and experience of smoking and alcohol are age-dependent (see online Supplementary Table S1), these characteristics were also different among those with missing data.

**Table 1.** Participant characteristics in those eligible and included in the analyses of first pregnancies (*n* 2921*) and those eligible but excluded from analyses due to missing data

	Eligible and included				Eligible but missing data				*P* †
	*n*	%	Mean	sd	*n*	%	Mean	sd	
At diet assessment (adolescence)									
Age (years)	2921		16·0	1·7	690		16·5	2·2	<0·001
WHO BMI (z-score)	2921		0·17	0·89	377		0·13	0·93	0·37
Education plans (higher education)	2921	34			567	34			0·9
Smoking (ever)	2921	59			623	61			0·29
Alcohol (ever)	2921	84			570	88			0·021
Snus use (ever)	2921	4·8			639	6·0			0·23
At first pregnancy									
Maternal age at birth (years)	2921		25·9	4·4	690		25·4	4·8	0·005
Smoking at beginning of pregnancy (yes)	2471	16·6			526	18·4			0·32
Dietary exposures (adolescence)‡									
Healthy foods index (0–70)	2921		31	12	690		27	15	<0·001
Fibre index (0–42)	2921		21	10	690		18	11	<0·001
Fruit and vegetable index (0–28)	2921		12	7	690		10	8	<0·001
Unhealthy foods index (0–56)	2921		11	6	690		10	7	0·11
Daily breakfast (yes)	2921	64			629	63			0·6
Daily lunch (yes)	2921	61			620	59			0·31
Daily dinner (yes)	2921	71			629	70			0·5
Outcomes (first pregnancy)									
Hypertensive disorder of pregnancy:									
Normotensive	2921	92				94·5			
Hypertensive		2·36			620	1·61			0·10
Pre-eclampsia/eclampsia		5·61				3·87			
Preterm delivery	2921	6·50			579	8·46			0·09

* Based on analysis of first pregnancies.

† *P*-values are from a *t* test for continuous variables and a *χ* ^2^ test for categorical variables.

‡ The values for the dietary exposures are obtained by summing the frequency of intake of components that comprise each index.

### Associations between adolescent exposures and potential confounders

Table 2 shows that those with healthier diets during adolescence were on average more likely to have plans for higher education and less likely to have ever smoked or used alcohol or chewed tobacco. Their first pregnancies also occurred on average at a slightly older age, and they were less likely to be smoking at conception. Similar patterns were seen among those who reported eating lunch daily during adolescence (see online Supplementary Table S2)

**Table 2.** Participant characteristics according to percentile-based categories of the healthy food index (dietary exposure)

	Healthy diet index (adolescence)			
	Lowest third (<24) *n* =	Middle third (24–35) *n* =	Highest third (>35) *n* =	*P*
At diet assessment:				
Age (years)	16·1	16·1	15·9	0·01
WHO BMI (z-score)	0·20	0·18	0·15	0·46
Education plans (higher education)	29·3 %	34·7 %	37·8 %	0·001
Smoking (ever)	67·1 %	57·7 %	51·8 %	<0·001
Alcohol (ever)	87·2 %	85·7 %	79·9 %	<0·001
Snus use (ever)	6·0 %	3·6 %	5·2 %	0·035
At birth*:				
Maternal age (years)	25·4	25·9	26·4	<0·001
Smoking at beginning of pregnancy (yes)	20·0 %	17·1 %	13·0 %	0·001

* Characteristics at first included pregnancy.

### Associations among dietary and meal exposures

Online Supplementary Tables S3 and S4 show the associations between the dietary and meal habit exposure variables. The healthy diet, fruit and vegetable, and fibre indexes shared strong correlations (range of r: 0·74–0·89), while the unhealthy index shared little correlation (range of r: −0·11 to −0·05). The daily meal habit exposures were weakly concordant with each other (range of kappa: 0·21–0·37). On average, adolescents who reported regular meal habits also had higher scores on the healthy diet, fruit and vegetable, and fibre indexes.

### Associations between adolescent diet and pregnancy outcomes

**Fig. 2.**
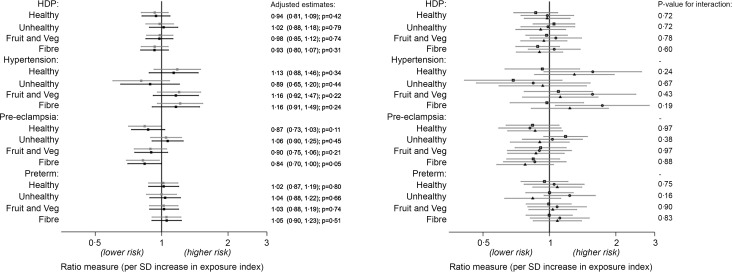
Association between diet indexes and hypertensive disorders and preterm birth in the first pregnancy (*n* 2921). Estimates are OR for HDP and preterm birth outcome (logit models) and relative risk ratios for hypertension and pre-eclampsia (multinomial logit models). Left plot: no age interaction (grey: crude; black: adjusted*). Right plot: unadjusted associations stratified by age of diet assessment (spit by tertiles: squares 13–15·1 years; circles 15·2–16·9 years; triangles 17–19 years). *Adjusted for age, WHO BMI z-score, alcohol (ever), smoking (ever), snus use (ever) and education plans at diet assessment, and maternal age at birth. HDP, hypertensive disorders of pregnancy.

Figure 2 shows the associations between the adolescent dietary pattern indexes and outcomes in the first pregnancy, while Fig. S1 (see online Supplementary information) shows the same results but for risk in any pregnancy. There was a weak suggestion of an association between adolescent fibre intake and pre-eclampsia – an sd higher fibre index score was associated with a 16 % reduction in risk of pre-eclampsia in the first pregnancy. This association was weaker when examining risk in any pregnancy (–12 %, *P* = 0·10) and was attenuated in the sensitivity analyses that included all available data in the unadjusted model (risk ratio in complete cases: 0·82, *P* = 0·02; risk ratio using all available data: 0·87, *P* = 0·08 – see online Supplementary Fig. S2). There was little evidence of any other association between the diet indexes and outcomes. Likewise, there was little suggestion of any difference or any consistent qualitative pattern in the associations stratified by the age at which diet was assessed. Adjusting for potential confounders generally attenuated estimates slightly towards the null.

**Fig. 3.**
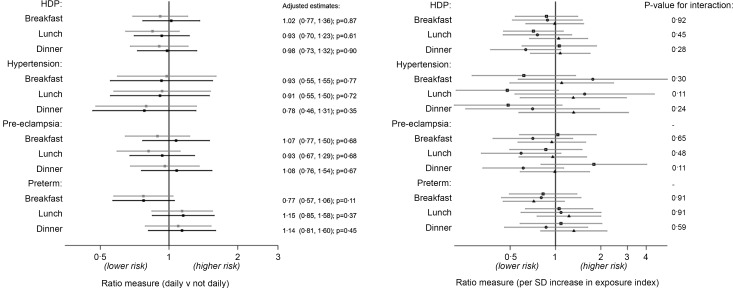
Association between meal patterns and hypertensive disorders and preterm birth in the first pregnancy (*n* 2921). Estimates are OR for HDP and preterm birth outcome (logit models) and relative risk ratios for hypertension and pre-eclampsia (multinomial logit models). Left plot: no age interaction (grey: crude; black: adjusted*). Right plot: unadjusted associations stratified by age of diet assessment (spit by tertiles: squares 13–15·1 years; circles 15·2–16·9 years; triangles 17–19 years). *Adjusted for age, WHO BMI z-score, alcohol (ever), smoking (ever), snus use (ever) and education plans at diet assessment, and maternal age at birth. HDP, hypertensive disorders of pregnancy.

The results for adolescent meal patterns are presented in the same format for the outcomes in the first pregnancy (Fig. 3) and for the outcomes in any pregnancy (see online Supplementary Fig. S3). There was little evidence to support an effect of meal patterns on HDP or preterm birth outcomes in the unstratified analyses. However, in the age-stratified analysis, there was a weak suggestion that the earlier period of adolescence examined (13–15 years) might be a sensitive period with regular lunch associated with a lower risk of later hypertension. In the sensitivity analysis using all available data, adolescents aged 13–15 years that reported consuming regular breakfast also had a lower risk of hypertension in pregnancy (see online Supplementary Fig. S4).

## Discussion

### Summary of findings

This is the first study to estimate the association between adolescent diet and meal patterns and risk of hypertensive disorders in pregnancy and preterm birth. We found a slightly lower risk of pre-eclampsia in the first pregnancy among those women who had reported a higher fibre intake during adolescence, although this result was somewhat dependent on the reproductive window examined and was not robust in sensitivity analyses to explore bias by missing data. Meal habits showed an age-dependent association with hypertension – those reporting eating regular meals from 13 to 15 years, particularly breakfast and lunch, had a lower risk of hypertension.

### Comparison with other studies and mechanisms related to fibre and pre-eclampsia

The associations between fibre intake and pre-eclampsia are in line with Shoenaker *et al.* (2015) who reported inverse associations between Mediterranean-style dietary pattern (characterised by vegetables, legumes, nuts, tofu, rice, pasta, rye bread, red wine and fish) and risk of developing HDP confined to GH (quartile 4 compared with quartile 1: RR, 0·58; 95 % CI, 0·42, 0·81)^(19)^. It also concords with Hillesund *et al.* 2014 who reported an association between a healthy pre-pregnancy diet score and pre-eclampsia, the score reflecting fibre intake especially from fruit and vegetables^(8)^.

Fibre index in the present study was derived from frequency of fruit, vegetable and wholegrain bread intake. So what does the fibre index represent in terms of nutritional value of relevance to pre-eclampsia? A higher intake of dietary fibre aids in weight maintenance. Fibre also lowers serum TAG and LDL-cholesterol, known to increase the risk of hypertension and CVD. A narrative review by Perry *et al.* (2022) highlighted that consuming a high-fibre diet (25–30 g/d) may reduce the risk of pre-eclampsia through beneficial effects on inflammation, blood lipids and blood pressure^(33)^. Dietary fibre intake is also a marker of foods with high nutritional value, with wholegrains containing Mg, Se, folate and other B-vitamins. The other food group rich in fibre is fruits and vegetables with ample amounts of antioxidants and anti-inflammatory potential. Dietary fibre also acts as a prebiotic through promoting the growth and abundance of beneficial large intestine bacteria^(34)^. Intake of probiotics during pregnancy has been shown to reduce the risk of pre-eclampsia^(35)^.

### Comparison with other studies and mechanisms related to meal habits and gestational hypertension

Those reporting eating regular meals in mid-adolescence (13–15 years), particularly breakfast and lunch, had a lower risk of hypertension compared with those with a similar exposure in later periods of adolescence. No other studies have investigated this association. What does eating breakfast and lunch daily *v*. less often imply? In Norway, breakfast and lunch typically include wholegrain bread or cereal which are main sources of dietary fibre in the diet. In addition, regularly eating breakfast and lunch provides metabolic fuel and critical nutrients for growth and accrual of body mass in this life stage. Regular meals may also prevent overeating and obesity, although the evidence on causality of this association is inconsistent^(36)^.

The observed association between dietary behaviour (fibre intake, and daily breakfast and lunch) among 13–15-year-olds and reduced odds of pre-eclampsia and GH, respectively, many years later, could be explained by habits in early adolescence tracking into adulthood. Another explanation of the finding confined to those who reported diet at 13–15 years could be that mid-adolescence is a biologically sensitive phase in girls regarding meal habits and related diet quality and later reproductive health.

### Strengths and limitations

Strengths of our study include the unique sample containing over 7000 births with prospectively measured diet and a long follow-up, enabling assessment of diet in adolescence in relation to not only first pregnancy but also the entire reproductive window for most mothers. Further, the diagnosis of pre-eclampsia and GH was based on hospital medical records from the Medical Birth Registry of Norway and is objectively verified. Disentangling cause from association is notoriously troublesome in observational nutrition research; in this respect, our analysis stratified by age of exposure offered an additional test to help triangulate our findings. Nonetheless, there are still assumptions such as measurement error to be similar across ages that would affect the validity of these comparisons.

Among limitations are the quality of the dietary data, which lacks resolution due to the small number of food items included in the questionnaire. Information on fish, meat, fats, and more information on fruits, berries and vegetables variety are also important aspects to consider. The highest obtainable frequency of intake was twice or more per d. In addition to possible dilution of associations by random error, there is also likely to be differential measurement error caused by under-reporting of unhealthy dietary components by more at-risk groups such as those with high BMI^(37)^.

### Conclusions

Our results are the first to indicate an association between aspects of diet and dietary behaviour in mid-adolescence and subsequent HDP. While a healthy diet throughout life is well advocated, a better understanding of sensitive windows for dietary intervention in relation to aspects of maternal health is still needed. With regard to our findings, more evidence is needed to replicate the results and to disentangle causality. Such studies will need larger samples with more detailed measures of diet and confounders and will exploit alternative designs with different sources of bias in order to triangulate findings.

## Supporting information

Wills et al. supplementary material Wills et al. supplementary material

## Supplementary material

For supplementary material/s referred to in this article, please visit https://doi.org/10.1017/S0007114524000746
