# Supplementary material for: Preconception diet in adolescence and its association with hypertensive disorders of pregnancy and preterm birth. Results from the HUNT study
Source: Br J Nutr. 2024 Apr 18;132(1):91–8. doi: 10.1017/S0007114524000746 (PMC7616499; doi:10.1017/S0007114524000746)
Supplement: Wills et al. supplementary material [file S0007114524000746sup001.docx]

**Supplementary Information**

*Preconception diet in adolescence and its association with hypertensive disorders of pregnancy and preterm birth. Results from The HUNT study.*

*Wills AK, Hillesund ER, Van Lippevelde W, Barker M, Vik FN, Øverby NC.*

Table S1. Relationship between age of diet assessment and maternal age and smoking status at the start of pregnancy

|  | Age at diet assessment | | | p-value |
| --- | --- | --- | --- | --- |
|  | 13 to 15.1y  (n=1017) | 15.2 to 16.9y  (n=942) | 17 to 19y  (n=962) |  |
| At diet assessment |  |  |  |  |
| WHO BMI (z-score) | 0.15 | 0.14 | 0.24 | 0.03 |
| Education plans (Higher education) | 13.7% | 30.8% | 59.0% | 0.001 |
| Smoking (ever) | 44.4% | 62.5% | 69.7% | <0.001 |
| Alcohol (ever) | 66.9% | 91.0% | 96.2% | <0.001 |
| Snus use (ever) | 3.3% | 4.1% | 7.0% | <0.001 |
| At birth |  |  |  |  |
| Maternal age (years) | 25.4 | 25.8 | 26.6 | 0.14 |
| Smoking at beginning of pregnancy (yes)* | 17.3% | 17.6% | 15.0% | 0.32 |

Table S2. Participant characteristics* according to lunch habits (meal habits exposure)

|  | Daily Lunch | |  |
| --- | --- | --- | --- |
|  | No | Yes | p-value |
| At diet assessment: |  |  |  |
| Age (years) | 16.1 | 16.0 | 0.1 |
| WHO BMI (z-score) | 0.27 | 0.12 | <0.001 |
| Education plans (Higher education) | 34.6% | 33.9% | 0.7 |
| Smoking (ever) | 67.5% | 52.8% | <0.001 |
| Alcohol (ever) | 89.6% | 80.1% | <0.001 |
| Snus use (ever) | 6.5% | 3.7% | 0.035 |
| At birth: |  |  |  |
| Maternal age (years) | 25.6 | 26.1 | <0.001 |
| Smoking at beginning of pregnancy (yes)* | 21.1% | 13.7% | 0.001 |

**^♯^** Characteristics at first included pregnancy

Table S3. Relationship between dietary indexes* and between meal habits†

|  | Healthy Index | Unhealthy Index | Fruit & Vegetable Index | Fibre Index | Daily breakfast | Daily Lunch |
| --- | --- | --- | --- | --- | --- | --- |
| Healthy Index | 1 |  |  |  |  |  |
| Unhealthy Index | -0.11 | 1 |  |  |  |  |
| Fruit & Vegetable Index | 0.75 | -0.05 | 1 |  |  |  |
| Fibre Index | 0.89 | -0.09 | 0.87 | 1 |  |  |
| Daily breakfast |  |  |  |  | 1 |  |
| Daily Lunch |  |  |  |  | 0.37 | 1 |
| Daily Dinner |  |  |  |  | 0.21 | 0.24 |

*correlation coefficients

†kappa coefficients

Table S4. Relationship between dietary Indexes and meal habits. The table shows the mean standardised score for each of the dietary indexes stratified by regular consumption of meals. In brackets is the standard deviation of the standardised score which by construction would take a value of 1 in the whole sample.

|  | Daily breakfast | | Daily Lunch | | Daily Dinner | |
| --- | --- | --- | --- | --- | --- | --- |
|  | No | Yes | No | Yes | No | Yes |
| Healthy Index | -0.28  (0.94) | 0.24  (0.93) | -0.22  (0.92) | 0.23  (0.94) | -0.25  (0.92) | 0.18  (0.95) |
| Unhealthy Index | 0.13  (0.99) | -0.04  (0.90) | 0.08  (0.91) | -0.02  (0.95) | 0.05  (1.01) | 0.01  (0.90) |
| Fruit & Vegetable Index | -0.08  (0.95) | 0.10  (0.99) | -0.10  (0.92) | 0.12  (1.01) | -0.20  (0.90) | 0.13  (1.0) |
| Fibre Index | -0.19  (0.93) | 0.17  (0.96) | -0.17  (0.91) | 0.18  (0.98) | -0.24  (0.91) | 0.15  (0.97) |


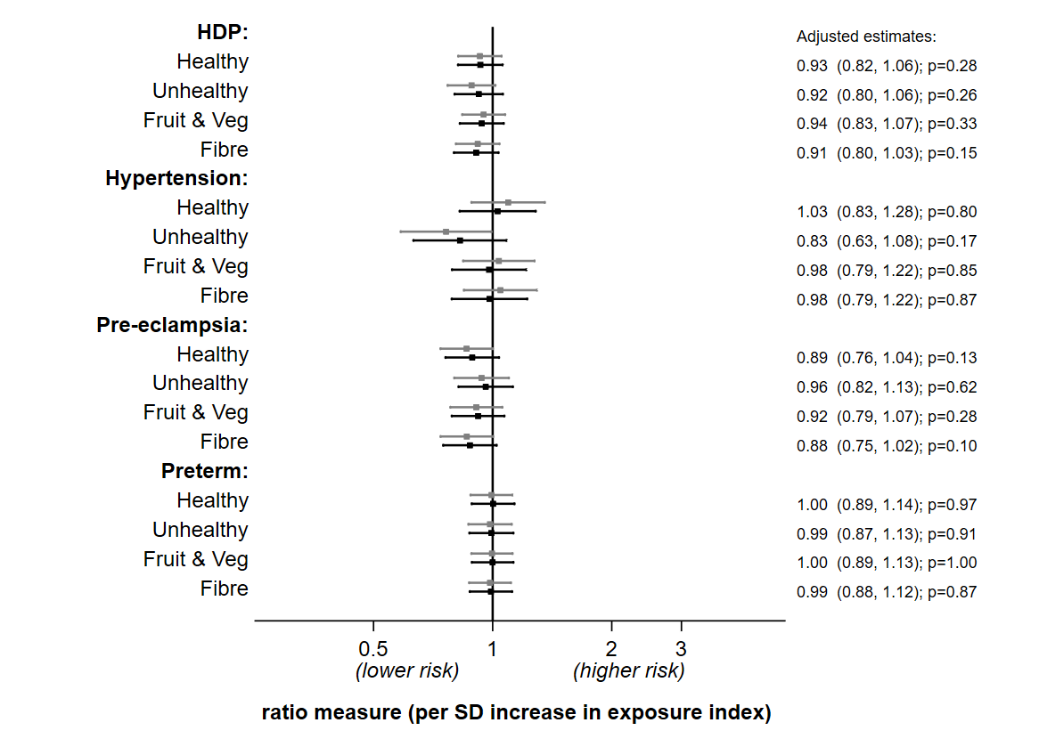

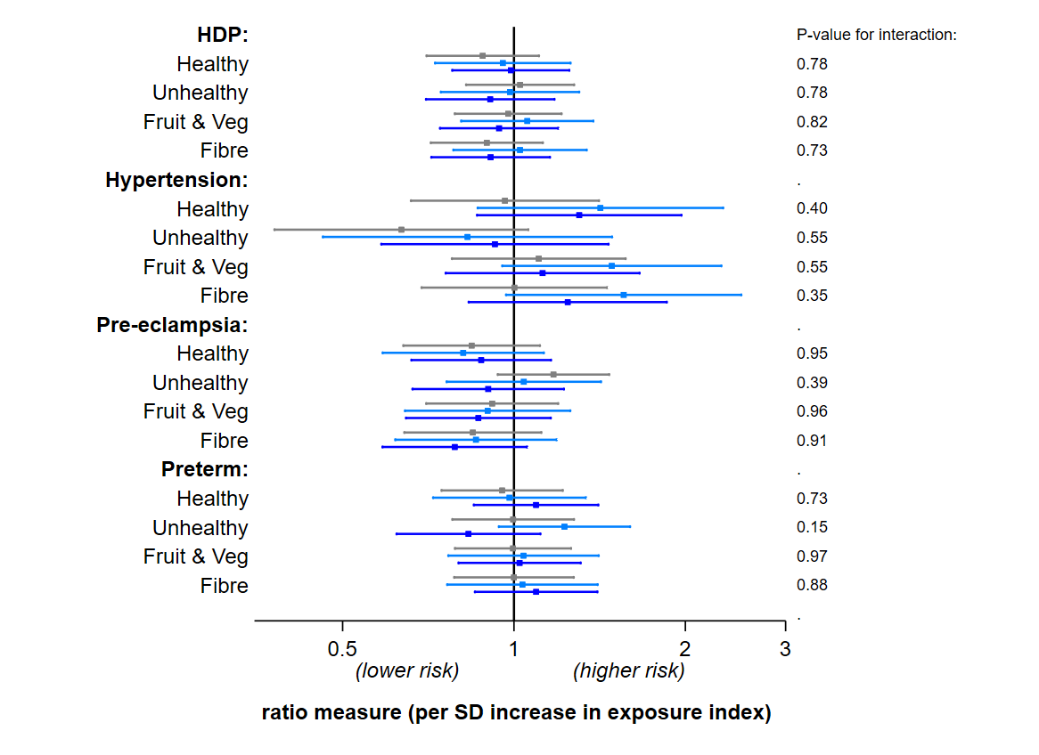


Figure S1. Association between diet indexes and hypertensive disorders and preterm birth in ANY pregnancy (n=2979).

Estimates are odds ratios for HDP and preterm birth outcome (logit models) and relative risk ratios for hypertension and pre-eclampsia (multinomial logit models). Left plot: no age interaction (grey: crude; black: adjusted*). Right plot: unadjusted associations stratified by age of diet assessment (spit by tertiles: grey 13-15.1y; light blue 15.2-16.9y; blue 17-19y).

*adjusted for age, WHO BMI z-score, alcohol (ever), smoking (ever), snus use (ever) and education plans at diet assessment, and maternal age at birth.


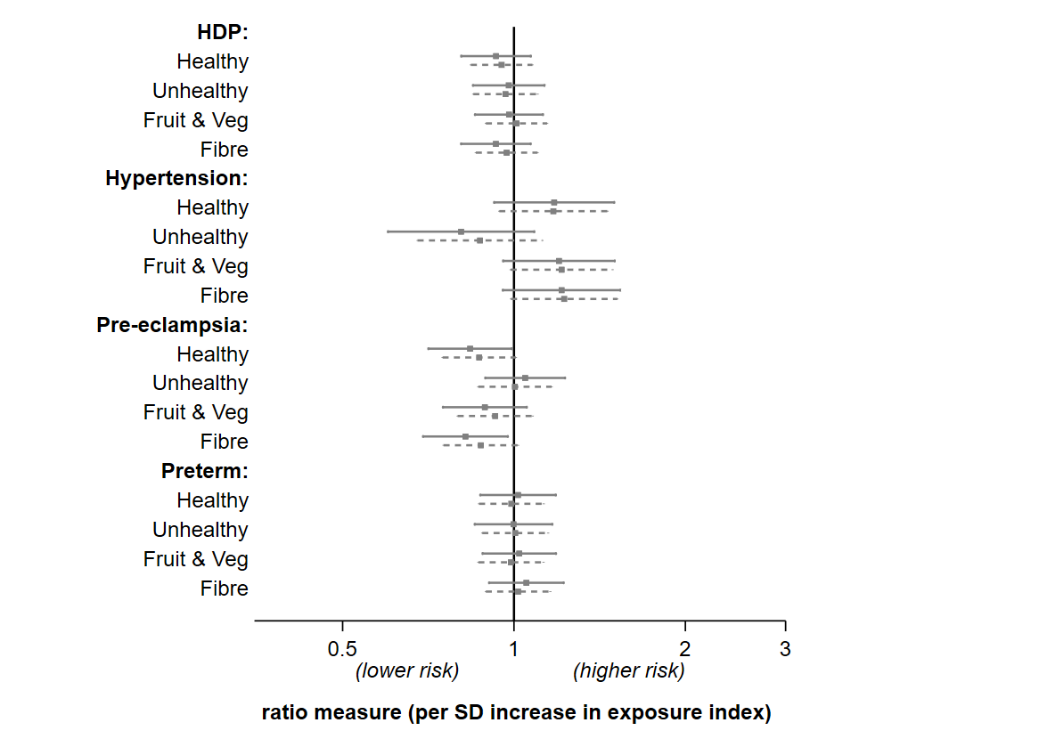

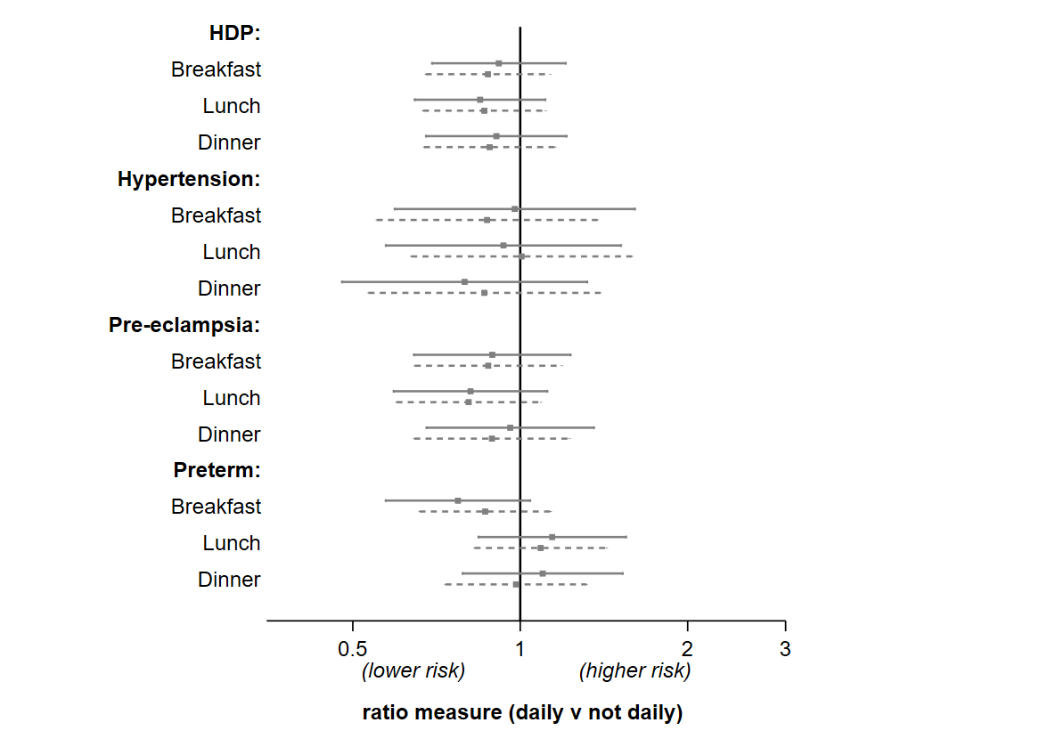


Figure S2. Sensitivity analysis: Association of diet indexes and meal habits with hypertensive disorders and preterm birth in the first pregnancy using all available data in crude (unadjusted) models.

Estimates are odds ratios for HDP and preterm birth outcome (logit models) and relative risk ratios for hypertension and pre-eclampsia (multinomial logit models). Left plot: diet indexes (solid: complete cases – n=2921; dash: all available data, n HDP=3541, n preterm=3500). Right plot: Meal patterns (solid: complete cases – n=2921; dash: all available data, n=3439 to 3481)


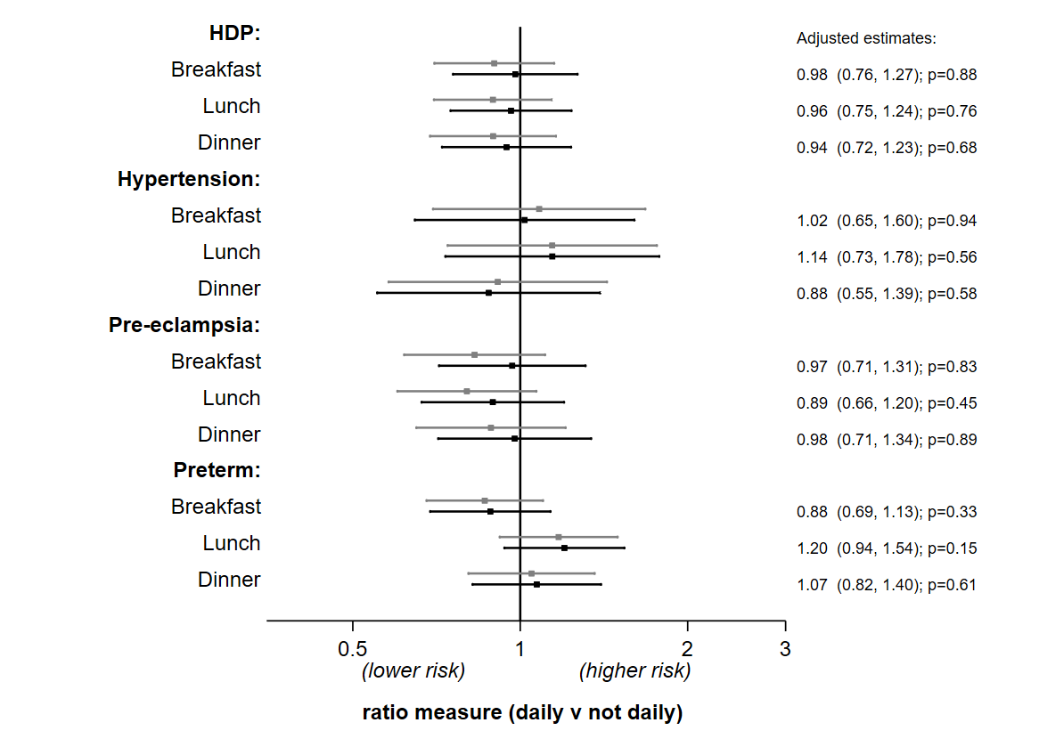

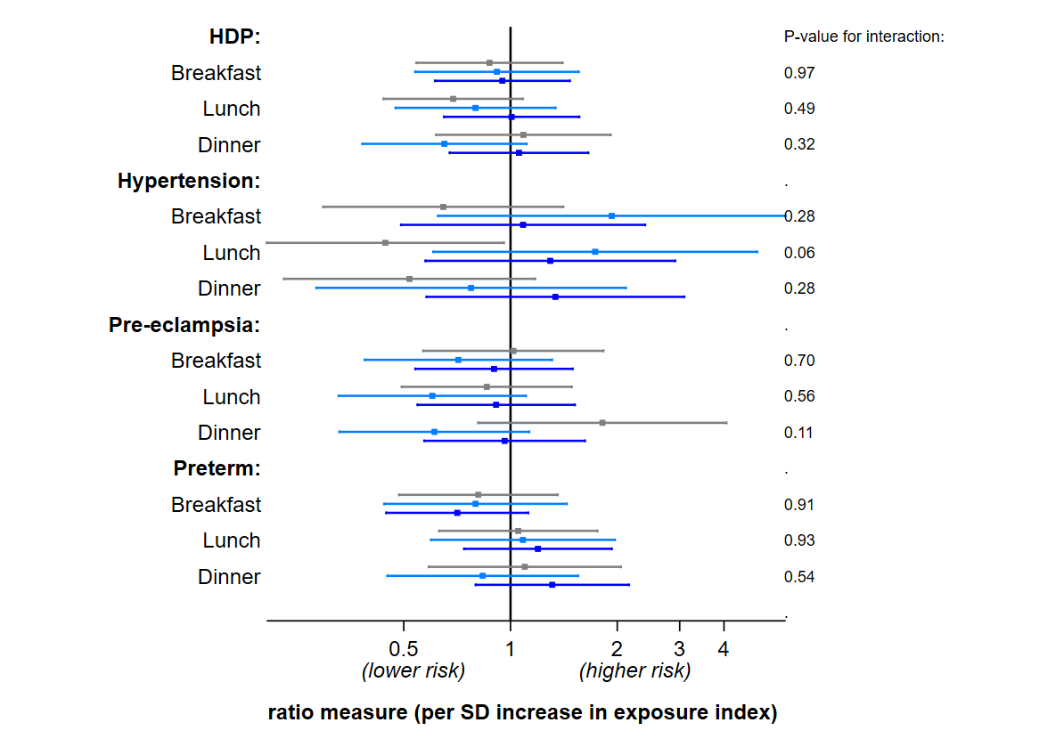


Figure S3. Association between meal patterns and hypertensive disorders and preterm birth in ANY pregnancy (n=2979).

Estimates are odds ratios for HDP and preterm birth outcome (logit models) and relative risk ratios for hypertension and pre-eclampsia (multinomial logit models). Left plot: no age interaction (grey: crude; black: adjusted*). Right plot: unadjusted associations stratified by age of diet assessment (spit by tertiles: grey 13-15.1y; light blue 15.2-16.9y; blue 17-19y).

*adjusted for age, WHO BMI z-score, alcohol (ever), smoking (ever), snus use (ever) and education plans at diet assessment, and maternal age at birth.


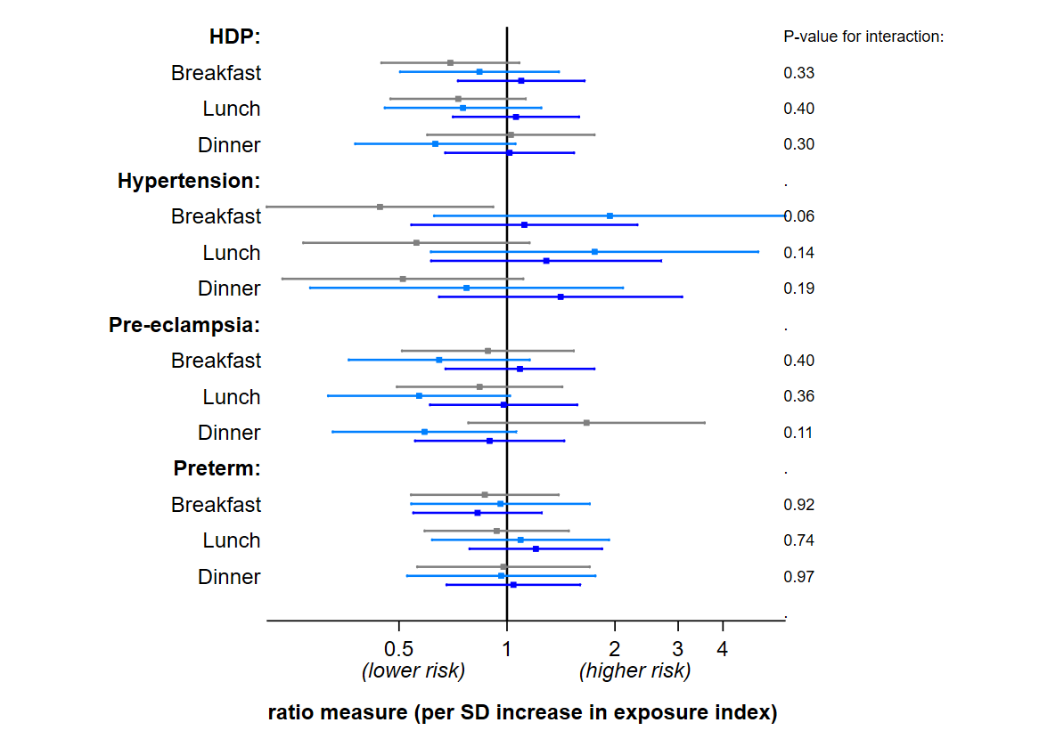

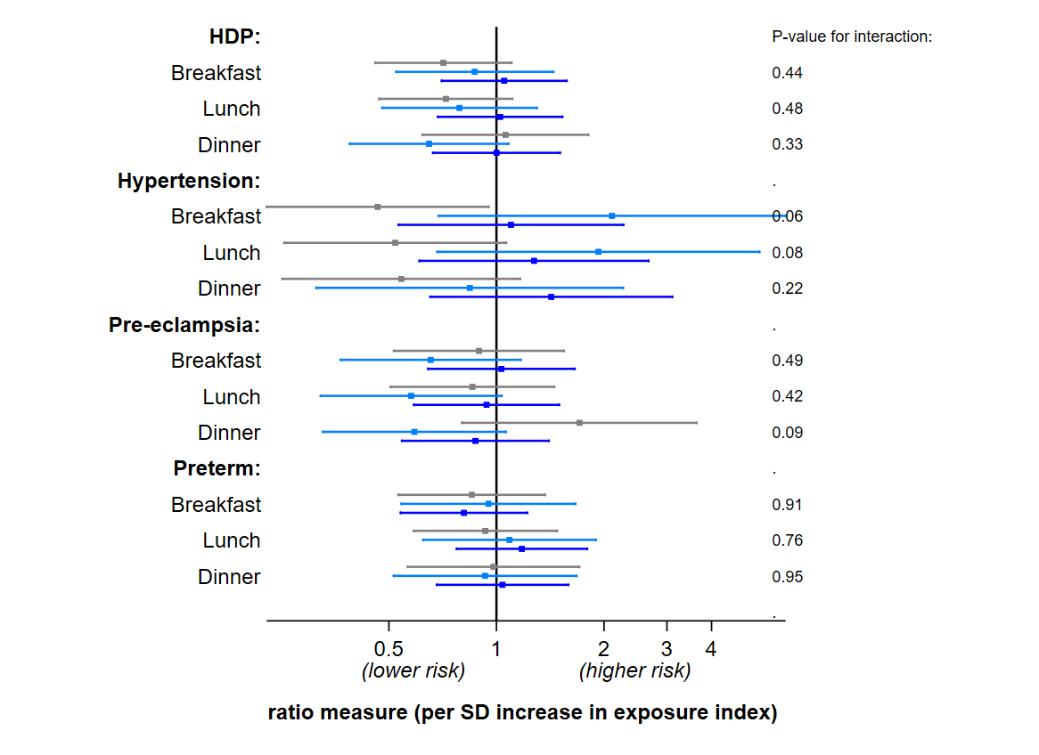


Figure S4. Sensitivity Analysis. Association between meal patterns and hypertensive disorders and preterm birth in the first pregnancy (left plot ,n=3430-3481) and any pregnancy (right plot, n=3500-3550) using all available data stratified by age of assessment (spit by tertiles: grey 13-15.1y; light blue 15.2-16.9y; blue 17-19y).

Estimates are odds ratios for HDP and preterm birth outcome (logit models) and relative risk ratios for hypertension and pre-eclampsia (multinomial logit models).
